# Serologic and PCR testing of persons with chronic fatigue syndrome in the United States shows no association with xenotropic or polytropic murine leukemia virus-related virus

**DOI:** 10.1186/1742-4690-8-S1-A232

**Published:** 2011-06-06

**Authors:** William M Switzer, Hongwei Jia, HaoQiang Zheng, Shaohua Tang, Rebecca A Garcia, Brent C Satterfield

**Affiliations:** 1 Division of HIV/AIDS Prevention, CDC, Atlanta, Georgia, 30333, USA; 2 Cooperative Diagnostics, LLC, Greenwood, SC, 29646, USA

## Background

In 2009, a newly discovered human retrovirus, xenotropic murine leukemia virus (MuLV)-related virus (XMRV), was reported by Lombardi et al. in 67% of persons from the US with chronic fatigue syndrome (CFS) by PCR detection of gag sequences. Although six subsequent studies have been negative for XMRV, CFS was defined more broadly using only the CDC or Oxford criteria and samples from the US were limited in geographic diversity, both potentially reducing the chances of identifying XMRV-positive CFS cases. A seventh study recently found polytropic MuLV sequences, but not XMRV, in a high proportion of persons with CFS from the US. Many explanations have been proposed for these discrepant results including differences in patient populations and methods used to classify CFS. Thus, more work is necessary to investigate the prevalence of these murine retroviruses in persons with CFS.

## Materials and methods

We tested blood specimens from 45 CFS cases and 42 persons without CFS from over 20 states in the United States. CFS patients were diagnosed using the CDC 1994 research case definition and the majority (31/45, (69%)) had a minimum of 6 months of post-exertional malaise and a high degree of disability, similar to the CFS patients in the Lombardi et al study. Plasma specimens were tested for antibodies using a new Western blot assay that utilizes purified XMRV as antigen. Whole blood DNA samples were tested using two generic XMRV/MuLV PCR tests in the polymerase region and one for gag sequences. Plasma from the CFS patients was also tested for viral RNA using a nested gag PCR test and a real-time PCR test that generically detects XMRV/MuLV gag sequences.

## Results

We found no evidence of either XMRV or MuLV in any of the 45 CFS cases or in the 42 persons without CFS, using a comprehensive PCR and serologic testing strategy.

## Conclusions

Our findings are consistent with previous negative reports and do not support an association of XMRV or MuLV in the majority of CFS cases across the US.

